# Protective Effects of Proanthocyanidin-Rich Fraction from Red Rice Germ and Bran on Lung Cell Inflammation via Inhibition of NF-κB/NLRP3 Inflammasome Pathway

**DOI:** 10.3390/nu15173793

**Published:** 2023-08-30

**Authors:** Warathit Semmarath, Kamonwan Srisawad, Punnida Arjsri, Sonthaya Umsumarng, Supachai Yodkeeree, Sansanee Jamjod, Chanakan Prom-u-thai, Pornngarm Dejkriengkraikul

**Affiliations:** 1 Akkhraratchakumari Veterinary College, Walailak University, Nakhon Si Thammarat 80160, Thailand; warathit.se@wu.ac.th; 2 Department of Biochemistry, Faculty of Medicine, Chiang Mai University, Chiang Mai 50200, Thailand; k.srisawad@gmail.com (K.S.); punnida_dream@hotmail.com (P.A.); yodkeelee@hotmail.com (S.Y.); 3 Anticarcinogenesis and Apoptosis Research Cluster, Faculty of Medicine, Chiang Mai University, Chiang Mai 50200, Thailand; 4 Division of Veterinary Preclinical Sciences, Department of Veterinary Biosciences and Veterinary Public Health, Faculty of Veterinary Medicine, Chiang Mai University, Chiang Mai 50200, Thailand; sonthaya.u@cmu.ac.th; 5 Lanna Rice Research Center, Chiang Mai University, Chiang Mai 50200, Thailand; sansanee.cm@gmail.com (S.J.); chanakan.p@cmu.ac.th (C.P.-u.-t.); 6 Department of Plant and Soil Sciences, Faculty of Agriculture, Chiang Mai University, Chiang Mai 50200, Thailand

**Keywords:** red rice germ and bran, proanthocyanidin-rich fraction, anti-inflammation, NLRP3 inflammasome, NF-κB nuclear translocation

## Abstract

The activation of the NLRP3 inflammasome pathway during infectious pathogen-induced immunopathology can lead to chronic inflammation and various adverse health outcomes. Identification of functional foods with anti-inflammatory properties is crucial for preventing inflammation triggered by NLRP3 inflammasome activation. This study aimed to investigate the anti-inflammatory properties of a proanthocyanidin-rich fraction obtained from red rice germ and bran against lipopolysaccharide (LPS) and adenosine triphosphate (ATP)-induced condition in A549 lung cells. The proanthocyanidin-rich fraction from Yamuechaebia 3 red rice extract (YM3-PRF) was obtained using column chromatography with Sephadex LH20, and its total proanthocyanidin content was determined to be 351.43 ± 1.18 mg/g extract using the vanillin assay. A549 lung cells were pretreated with YM3-PRF at concentrations of 5–20 μg/mL prior to exposure to LPS (1 μg/mL) and ATP (5 nM). The results showed that YM3-PRF significantly inhibited the expression of inflammatory mRNAs (NLRP3, IL-6, IL-1β, and IL-18) and the secretion of cytokines (IL-6, IL-1β, and IL-18) in a dose-dependent manner (*p* < 0.05). Mechanistically, YM3-PRF exerted its anti-inflammatory effects by inhibiting NF-κB translocation and downregulating proteins associated with the NLRP3 inflammasome pathway (NLRP3, ASC, pro-caspase-1, and cleaved-caspase-1). These findings suggest that the proanthocyanidin-rich fraction from red rice germ and bran has protective effects and may serve as a potential therapeutic option for chronic inflammatory diseases associated with NLRP3 inflammasome activation.

## 1. Introduction

Diseases affecting the respiratory system, particularly lung diseases, remain significant causes of illness and mortality worldwide. These include lower respiratory tract infections, chronic obstructive pulmonary disease (COPD), and tuberculosis [1,2]. Despite their prevalence, effective treatments for respiratory diseases are still limited, primarily due to a lack of complete understanding regarding their pathogenesis and challenges in adequately controlling or suppressing the inflammatory processes [3]. A key player in the immune system that contributes to various chronic inflammatory conditions involving the lungs is the NLRP3 inflammasome [4,5,6,7]. This molecular complex plays a crucial role in driving inflammatory responses associated with conditions such as acute respiratory distress syndrome (ARDS), hyperresponsive lung inflammation due to bacterial infections, COVID-19-related inflammation, and age-related lung inflammation. Understanding the role of NLRP3 inflammasomes in these diseases could pave the way for targeted therapies and improved management of respiratory system disorders.

Chronic inflammation resulting from the activation of the NLRP3 inflammasome can have significant consequences on the lungs, leading to irreversible architectural changes that disrupt normal physiological function [8]. Studies in mice have revealed that the absence of the inflammasome pathway adaptor protein (ASC-/-Mice) prevents the development of pulmonary fibrosis, a condition characterized by inflammatory and fibrotic alterations in the lung tissue, even when exposed to inflammatory agents like Bleomycin [9]. Furthermore, age plays a crucial role in the immune response to infections, particularly in individuals aged 65 and above. In this elderly population, infections caused by bacteria or viruses can trigger a more severe inflammation of lung tissue through the activation of NLRP3 inflammasome, resulting in a higher mortality rate compared to other age groups [10,11,12]. Considering these findings, targeting the NLRP3 inflammasome pathway presents a potential therapeutic approach to reduce inflammation and halt the progression of lung fibrosis during respiratory system infections. Such interventions could hold promise in mitigating the severity of viral infections in the lung and potentially lead to decreased mortality rates. Nevertheless, further research is needed to fully understand the translational implications of these preclinical findings to human treatments. Additionally, staying up to date with the latest advancements in this field is essential to inform clinical decisions effectively.

In recent research, pigmented rice varieties such as black rice and red rice have drawn significant attention due to their potential health benefits. These rice types, specifically their outer layers comprising the germ and bran, have been found to exhibit remarkable anti-inflammatory characteristics attributed to their high content of anti-inflammatory phytochemical compounds [13,14]. Moreover, studies have indicated a positive correlation between pigment content and total antioxidant capacity in these rice grains, suggesting that the increase in pigments also enhances their antioxidant properties [14]. Among the pigmented rice varieties, red rice stands out as a prominent source of proanthocyanidins pigment, which has been associated with potent antioxidant, anti-inflammatory, anticancer, and immunomodulatory activities [15,16,17]. Previous investigations have demonstrated that red rice grain extracts show promising antimetastasis effects on invasive human breast carcinoma cells (MDA-MB231) and human fibrosarcoma HT1080 cell lines [18,19]. Additionally, the proanthocyanidin-rich fraction from red rice has exhibited the ability to inhibit cell proliferation and induce apoptosis in HepG2 cells [20]. In experimental models, treatment with proanthocyanidin-rich red rice extract effectively reduced inflammatory cell infiltration and histopathological damage in dextran sulfate sodium-induced colitis in mice. Furthermore, this treatment suppressed the production of proinflammatory cytokines, including TNF-α, IL-1β, and IL-6 [21]. Despite these promising findings, no research has yet explored the potential of the proanthocyanidin-rich fraction from red rice as a therapeutic option for managing chronic inflammatory diseases associated with NLRP3 inflammasome activation.

Therefore, the current study focuses on native red rice varieties sourced from the northern region of Thailand to formulate a proanthocyanidin-rich fraction from red rice germ and bran: firstly, to investigate the inhibitory effects of the extract on the activation of NLRP3 inflammasome in A549 lung cells induced by lipopolysaccharide (LPS) and adenosine triphosphate (ATP); secondly, the study aims to develop proanthocyanidins from high-quality native red rice as a dietary supplement, with the intention of alleviating the severity of inflammation and chronic inflammatory conditions. The findings from this study could pave the way for new and natural therapeutic approaches in managing inflammatory diseases and improving human health.

## 2. Materials and Methods

### 2.1. Chemical and Reagents

Dulbecco’s Modified Eagle Medium (DMEM) was purchased from Gibco (Grand Island, NY, USA). Fetal bovine serum (FBS) was purchased from Thermo Scientific (Waltham, MA, USA). Modified radioimmunoprecipitation assay (RIPA) lysis buffer, protease inhibitor cocktail, Coomassie Plus™ Protein Assay Reagent, and chemiluminescent immunoblotting reagent were obtained from Thermo Fisher Scientific (Rockford, IL, USA). QIAzol lysis reagent was purchased from Qiagen (Valencia, CA, USA). ReverTra Ace^®^ qPCR Master Mix was purchased from Toyobo Co., Ltd. (Osaka, Japan). SensiFAST^TM^ SYBR^®^ Lo-ROX Kit was purchased from Meridian Bioscience^®^ (Cincinnati, OH, USA). The MTT or 3-(4,5-dimethylthiazol-2-yl)-2,5 diphenyltetrazolium bromide dye and mouse anti-β-actin primary antibody were purchased from Sigma-Aldrich Company (St. Louis, MO, USA). Anti-NLRP3 primary antibody, anti-ASC primary antibody, anti-caspase-1 primary antibody, anti-NF-κB primary antibody, anti-PARP primary antibody, and goat horseradish peroxidase-conjugated anti-mouse- or anti-rabbit-IgG were obtained from Cell Signaling Technology company (Danvers, MA, USA).

### 2.2. Rice Materials

Red rice (*Oryza sativa* L.) variety Yamuechaebia 3 CMU (YM3), a nonglutinous rice, was procured from the Lanna Rice Research Center at Chiang Mai University, Thailand. The herbarium voucher specimen of YM3 (voucher specimen no. PNPRDU63022) was validated and certified by the herbarium at the Flora of Thailand, Faculty of Pharmacy, Chiang Mai University, Thailand. To prepare samples, the whole grains of red rice (YM3) were subjected to milling. The rice milling process involved using a rice dehusker and a rice milling machine (Kinetic (Hubei) Energy Equipment Engineering Co., Ltd., Wuhan, China). The milling process was carefully conducted to obtain the germ and bran components of the red rice, which are known to be rich in bioactive compounds, including proanthocyanidins.

### 2.3. Preparation of Proanthocyanidin-Rich Fraction from Red Rice Germ and Bran

Red rice germ and bran from the YM3 variety were utilized to extract the proanthocyanidin-rich fraction (YM3-PRF), following a previously described protocol [13,14]. Initially, 100 g of red rice germ and bran were soaked in 50% ethanol for 24 h to facilitate the extraction of bioactive compounds including proanthocyanidins. After soaking, the samples were filtered using filter paper to separate the ethanolic fractions from the solid residue. The ethanolic fractions were then concentrated by evaporating the ethanol using a rotary vacuum evaporator (BUCHI, Flawil, Switzerland). Next, the concentrated ethanolic fractions were subjected to partitioning with saturated butanol (1:1 ratio of butanol to deionized water) using a separating funnel. This step allowed the separation of different compounds present in the extract. The butanol fractions were further processed by evaporating the butanol and subsequently freeze-drying the remaining material to obtain a powdered form. For the purification of the proanthocyanidins, the YM3-buthanol fraction (2.5 g) was dissolved in methanol and loaded onto a Sephadex LH20 column (GE Healthcare Ltd., Little Chalfont, UK). Sequential elution was carried out using 70% methanol and 70% acetone solutions, enabling the separation of proanthocyanidin based on their properties. Total contents of proanthocyanidin in each fraction were determined by vanillin assay. The fractions displaying high concentrations of proanthocyanidin were collected and combined to concentrate the desired compounds. The combined fractions were then freeze-dried to produce the final proanthocyanidin-rich fraction (YM3-PRF) in powdered form. For the determination of the total proanthocyanidin content, the YM3-PRF powder was resuspended in dimethyl sulfoxide (DMSO) and subjected to a vanillin assay, a commonly used method for quantifying proanthocyanidin through its reaction with vanillin under acidic conditions.

### 2.4. Determination of Total Phenolic and Total Flavonoid Content

The total phenolic content of YM3 red rice extracts used in this study was assessed using a modified Folin–Ciocalteu assay, following established procedures [15]. In this method, a specific volume of the red rice extract was mixed with Folin–Ciocalteu reagent and allowed to undergo a reaction. After an appropriate incubation period, the absorbance of the resulting mixture was measured at 765 nm using a UV–visible spectrophotometer. To quantify the phenolic content, the absorbance values were compared with a standard curve generated from known concentrations of gallic acid (GA). The total phenolic content was expressed as milligrams of gallic acid equivalents per gram of extract (mg GAE/g extract).

For the determination of total flavonoid content, the red rice extracts were analyzed using the aluminum chloride (AlCl_3_) colorimetric assay, following a method previously described [16]. In this procedure, a specific volume of the red rice extract was mixed with aluminum chloride reagent, leading to the formation of a complex. The absorbance of the resulting mixture was then measured at 510 nm using a spectrophotometer. By comparing the absorbance values with a standard curve generated from known concentrations of catechin, the total flavonoid content was expressed as milligrams of catechin equivalents per gram of extract (mg CE/g extract). All measurements were performed in triplicate, and the results were reported as mean value standard deviations to ensure accuracy and reliability in the data analysis.

### 2.5. Quantification of Proanthocyanidin Content

The total content of proanthocyanidin in YM3-PRF was determined using a modified vanillin assay [14]. Briefly, 40 μL of YM3-PRF was mixed with 100 μL of 1% (*w/v*) vanillin in methanol. Subsequently, 100 μL of 9 M sulfuric acid (H_2_SO_4_) was added to the sample, and the mixture was incubated at 30 °C for 15 min. To quantify the proanthocyanidin content in the sample, spectrophotometry was performed, and the absorbance was measured at 490 nm. The absorbance values were then compared with a catechin standard curve. The amount of total proanthocyanidin content was expressed as milligrams of catechin equivalents per gram of YM3-PRF extract (mg CE/g extract). All analyses were conducted in triplicate to ensure accuracy, and the results were presented as mean values ± standard deviations.

### 2.6. ABTS and DPPH Assays for Antioxidant Properties

The antioxidant activity of the YM3-PRF was determined using ABTS assay, as has been previously described [17,18]. Absorbance was recorded at 734 nm using a spectrophotometer and compared to a calibration curve of the Trolox standard, which is the positive control for the experiment.

The DPPH radical-scavenging assay was determined as has been previously de-scribed [19]. Briefly, the 20 mL of various concentrations of YM3-PRF were mixed with 180 mL of freshly prepared DPPH methanolic solution and kept in the dark for 10 min. Then, absorbance was measured at 540 nm. Vitamin E was used as a positive control.

### 2.7. Cell Culture

The A549 lung epithelial cell line was obtained from the American Type Culture Collection (ATCC). The A549 cell normally has a polygonal shape and sheetlike pattern in normal monolayer culture, which is compatible with its epithelial origin [20]. The cells were maintained as a monolayer and cultured in Dulbecco’s Modified Eagle Medium (DMEM) supplemented with 10% FBS, 2 mM L-glutamine, 50 U/mL of penicillin, and 50 μg/mL of streptomycin. The cell culture was maintained in a 5% CO_2_ humidified incubator at 37 °C. When the cells created a monolayer and reached 70–80% confluency, the cells were harvested and plated for subsequent experimentation.

### 2.8. Cell Cytotoxicity Assay

To evaluate the cytotoxicity of YM3-PRF against A549 cells, a 3-(4,5-dimethylthiazol-2-yl)-2,5-diphenyltetrazolium bromide (MTT) assay was employed. A549 cells were seeded at a density of 3 × 10^3^ cells/well in a culture medium. When A549 cells created a monolayer and reached 70–80% confluency, the cells were then treated with increasing concentrations of YM3-PRF (ranging from 0–40 μg/mL) for 24 and 48 h. After the respective incubation periods, the cells were exposed to 10 μL of 0.5 mg/mL MTT solution in phosphate-buffered saline (PBS) and allowed to incubate for 4 h. The culture supernatant was then carefully removed, and the cells were resuspended with 200 μL of DMSO to dissolve the MTT formazan crystals. The absorbance of the resulting solution was measured at 540 and 630 nm using a UV–visible spectrophotometer. The assay was performed in triplicate for each concentration of YM3-PRF. Cell viability was determined by comparing the absorbance values to those of the control group and was expressed as a percentage relative to the control. By analyzing the data, the potential cytotoxic effects of YM3-PRF on A549 cells were assessed, providing insights into its impact on cell viability over the specified time and concentration ranges.

### 2.9. Determination of IL-6, IL-1β, and IL-18 Secretion by Enzyme-Linked Immunosorbent Assay (ELISA)

To assess the secretion of IL-6, IL-1β, and IL-18 cytokines in the culture medium, we employed an ELISA kit from BioLegend (San Diego, CA, USA) following the manufacturer’s instructions as previously outlined [21]. A549 cells (3 × 10^5^ cells/well) were seeded in a 6-well culture plate from Thermo Scientific (Waltham, MA, USA) and allowed to adhere overnight. When A549 cells created a monolayer and reached 70–80% confluency, the cells were pretreated with varying concentrations (0–20 μg/mL) of YM3-PRF for 24 h. Subsequently, the cells were exposed to 1 μg/mL lipopolysaccharide (LPS) for 6 h and 5 nM adenosine triphosphate (ATP) for 30 min to induce inflammatory responses. Following the respective incubation periods with LPS and ATP, the cultured medium from each well was collected for ELISA testing. After adding the samples to the 96-well ELISA microplate from Thermo Scientific (Waltham, MA, USA) that was coated with a specific capture antibody, the samples were incubated for 2 h. Following the incubation, the ELISA plates were washed 4 times with the 0.05% PBS-Tween. Subsequently, the detection antibody was added and left to incubate for 1 h. After the washing step, the substrate solution was added, and the reaction was stopped by 2N H_2_SO_4_. The absorbance was measured at 450 and 570 nm using a microplate reader (Sunrise, Tecan Trading AG, Männedorf, Switzerland). The cytokine releases were performed at least in triplicate and calculated by comparing with standard calibration curves for each cytokine.

### 2.10. Expression of IL-6, IL-1β, IL-18, and NLRP3 Genes by Reverse Transcription-Polymerase Chain Reaction (qRT-PCR) Analysis

To determine inflammatory gene expressions, A549 cells (3 × 10^5^ cells/well) were seeded in a 6-well plate and allowed to adhere overnight. When the cells created a monolayer and reached 70–80% confluency, the A549 cells were pretreated with YM3-PRF (0–20 μg/mL) for 24 h and then exposed to 1 μg/mL LPS for 6 h and 5 nM ATP for 30 min, respectively. To assess the gene expression levels, total mRNA from the treated cells was prepared using Qiazol reagent. The concentration and purity of the extracted RNA were determined using NanoDrop 2000/2000 c Spectrophotometers (Thermo Fisher Scientific, Waltham, MA, USA), ensuring that the RNA quality was suitable for further analysis. Next, we performed reverse transcription to convert the RNA into complementary DNA (cDNA) using a Mastercycler^®^ nexus gradient machine (Eppendorf, GA, Hamburg, Germany). Quantitative real-time PCR technique was determined using a qRT-PCR ABITM 7500 Fast & 7500 Real-Time PCR machine (Thermo Fisher Scientific, Waltham, MA, USA). Gene expressions were analyzed using QuantStudio 6 Flex real-time PCR system software v1.0 (Applied Biosystems, Waltham, MA, USA). The 2^−ΔΔCT^ method with normalization to GAPDH and controls was used for the calculation of results.

The primer sequences used for amplifying IL-6 were supplied from Bio Basic Canada Inc., Markham, ON, Canada. Nucleotide-binding oligomerization domain-like receptor containing pyrin domain 3 (NLRP3), interleukin-1beta (IL-1β), interleukin-18 (IL-18), and glyceraldehyde 3-phosphate dehydrogenase (GAPDH) primer sequences were supplied from Humanizing Genomics Macrogen, Geumcheongu, Seoul, Republic of Korea. All primer sequences used in this study are shown in Table 1.

### 2.11. Western Blotting Analysis of NLRP3 Inflammasome-Related Proteins

To examine the effects of YM3-PRF on NLRP3 inflammasome machinery proteins in LPS and ATP-induced inflammation, protein expression was assessed through Western blot analysis. Briefly, A549 cells (3 × 10^5^ cells/well) were seeded in a 6-well culture plate from Thermo Scientific (Waltham, MA, USA) and allowed to adhere overnight. When the cells created a monolayer and reached 70–80% confluency, A549 cells were pretreated with YM3-PRF (0–20 μg/mL) for 24 h and then exposed to 1 μg/mL LPS for 6 h and 5 nM ATP for 30 min, respectively. Then, cells were collected and lysed using RIPA buffer (Thermo Scientific, Waltham, MA, USA). The protein concentration was determined using the Bradford assay (Thermo Scientific, Waltham, MA, USA). The whole-cell lysate was subjected to 12% SDS-PAGE. Separated proteins were transferred into nitrocellulose membranes. Membranes were blocked with 5% BSA protein in 0.1% TBS-Tween. After that, the membranes were washed twice with 0.1% TBS-Tween. Then, membranes were further incubated overnight with the NLRP3 (1:1000), ASC (1:2000), or caspase-1 (1:1000) primary antibody at 4 °C. Next, the membranes were washed 5 times with 0.1% TBS-Tween followed by incubating with horseradish peroxidase-conjugated anti-mouse or rabbit-IgG (1:10,000), depending on the primary antibody, at room temperature for 2 h and were then washed 5 times with 0.1% the TBS-Tween. Bound antibodies were detected using the chemiluminescent detection system (Cytiva, Marlborough, MA, USA) and then exposed to the iBright™ CL-1500 imaging system (Thermo Fisher Scientific, Waltham, MA, USA). Equal values of protein loading were confirmed as each membrane was stripped and reprobed with an anti-β-actin antibody (1:10,000). Band density levels were analyzed using the “Measure” function in ImageJ 1.410 software (https://imagej.nih.gov/ij/, accessed on 19 December 2022).

### 2.12. NF-κB Nuclear Translocation

In order to prepare the nuclear extract for the determination of inhibitory effects on NF-κB nuclear translocation in LPS and ATP induction cells, the nuclear extract method was followed according to the previously described protocol [21]. Briefly, A549 cells were seeded at 1.5 × 10^6^ cells per culture dish overnight. When A549 cells created a monolayer and reached 70–80% confluency, then the cells were pretreated with YM3-PRF (0–20 μg/mL) for 24 h and then exposed to 1 μg/mL LPS for 6 h and 5 nM ATP for 30 min, respectively. Then, cells were collected and washed twice with ice-cold PBS. The cell pellet was suspended with cytoplasmic lysis buffer and gently mixed by a micropipette. The tubes were incubated on ice for 15 min and centrifuged at 10,000 rpm for 20 min. The supernatant was collected and was representative as the cytoplasmic extracts. The nuclear pellets were suspended in an ice-cold nuclear extraction buffer with intermittent vortex for 20 min. The nuclear extract was centrifuged at 10,000 rpm for 20 min, and the supernatant was collected and was representative of the nuclear extracts.

The nuclear and cytoplasmic extracts were determined for the protein concentration using the Bradford method. The expressions of NF-κB and PARP in the nuclear extracts and the expressions of NF-κB and β-actin in the cytoplasmic extracts were determined by Western blotting analysis using the specific antibodies to p65-NF-κB (1:2000). Equal values of nucleus protein loading were confirmed as each membrane was stripped and reprobed with an anti-PARP antibody (1:10,000). Equal values of cytoplasm protein loading were confirmed as each membrane was stripped and reprobed with an anti-β-actin antibody (1:10,000). Band density levels were analyzed using ImageJ 1.410 software (https://imagej.nih.gov/ij/, accessed on 19 December 2022).

### 2.13. Statistical Analysis

All data are presented as mean ± standard deviation (S.D.) values. Statistical analysis was analyzed with Prism version 8.0 software using independent t-test and one-way ANOVA with Dunnett’s test. Statistical significance was determined at * *p* < 0.05, ** *p* < 0.01, and *** *p* < 0.001.

## 3. Results

### 3.1. Phytochemical Characterization of YM3 Red Rice Extract

This study aimed to characterize the phytochemical constituents of YM3-butanol fractions obtained from red rice extract, focusing on total phenolic, total flavonoid, and total proanthocyanidin contents. Proanthocyanidin, previously identified as the active compound in red rice [13,14], was investigated in the YM3-butanol fractions. The total phenolic content was found to be 156.21 ± 10.82 mg GAE/g extract and the total flavonoid content was 116.07 ± 5.63 mg CE/g extract in the YM3-butanol fractions. As expected, this proanthocyanidin was identified in our YM3-butanol fractions. Proanthocyanidin was present in the YM3-butanol fractions with a concentration of 61.83 ± 4.93 mg/g extract. To further enhance the concentration of proanthocyanidin, chromatography using Sephadex LH20 was employed, resulting in the formation of a fraction referred to as “YM3-PRF”. The YM3-PRF displayed 2.31 of %yield and a higher concentration of proanthocyanidin, measuring 351.43 ± 1.18 mg/g extract. Consequently, the YM3-PRF was selected for further investigation regarding its anti-inflammatory effects on LPS + ATP-induced inflammation in A549 lung epithelial cells, with a focus on the inhibition of the NLRP3 inflammasome pathway.

### 3.2. Antioxidant Activities of YM3-PRF

The phytochemicals screening of YM3-PRF had given the perspective for choosing a candidate fraction for further bioactivity experiments. Additionally, phytochemical contents in plant extract could be responsible for the antioxidant properties. Thus, we further investigated the antioxidant activities of the YM3-PRF. As is shown in Table 2, the antioxidant activity of YM3-PRF was assessed using two distinct chemical-based antioxidant methods (ABTS and DPPH assays). As expected, the DPPH and ABTS assays showed that YM3-PRF exhibited strong antioxidants activity with an inhibitory concentration at 50% (IC_50_) of 1.75 ± 0.45 µg/mL (for the ABTS assay) which was significantly lower than the positive control, Trolox (*p* < 0.01). For the DPPH assay, YM3-PRF exhibited strong antioxidants activities with the IC_50_ of 9.14 ± 1.01 µg/mL, which was significantly lower than the positive control, vitamin E (*p* < 0.01). These results indicated that YM3-PRF provided a high amount of phytochemicals content and indeed possessed strong antioxidant activities.

### 3.3. Cytotoxicity of YM3-PRF on A549 Cells

**Figure 1:**
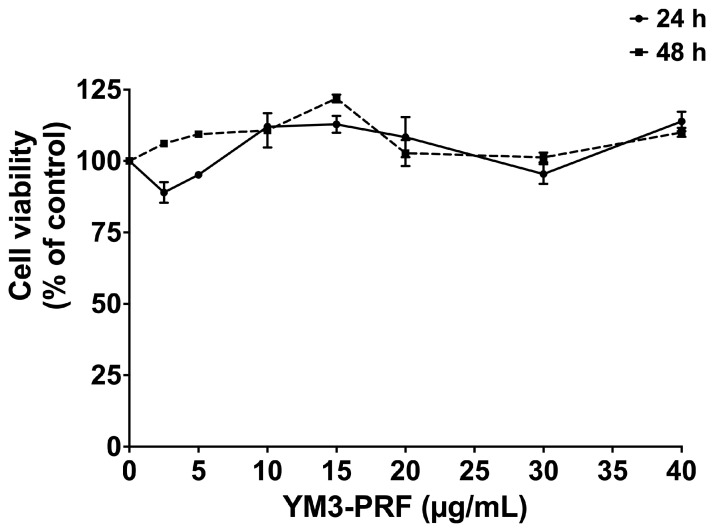
Cytotoxicity assessment of YM3-PRF on A549 lung cells. A549 cells (at a density of 3 × 10^3^ cells/well) were exposed to varying concentrations of YM3-PRF ranging from 0 to 40 μg/mL for 24 h and 48 h. The cell viability was determined using the MTT assay. The presented data represent the mean ± S.D. values obtained from a minimum of three independent experiments.

Prior to investigating the anti-inflammatory properties of YM3-PRF extract, we conducted a comprehensive evaluation of its cytotoxicity on A549 cells. The MTT assay was employed to assess cell viability, as illustrated in Figure 1. At concentrations ranging from 0–40 μg/mL, YM3-PRF extract displayed no cytotoxic effects on A549 cells following 24 h and 48 h treatments. These results indicate that the YM3-PRF extract is well-tolerated by A549 cells within the tested concentration range and treatment durations.

### 3.4. Effect of YM3-PRF on the Inhibition of Proinflammatory Cytokine Releases (IL-6, IL-1β, and IL-18) in LPS + ATP-Induced A549 Cells

**Figure 2:**
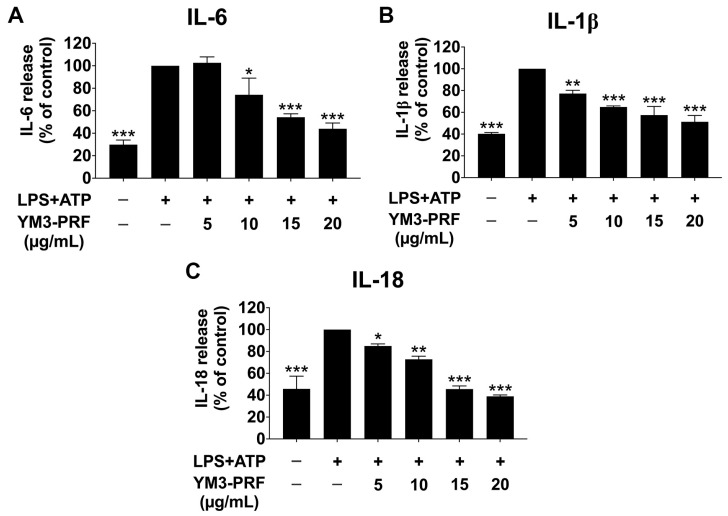
Inhibitory effects of YM3-PRF on proinflammatory cytokine release in LPS and ATP-induced A549 cells. A549 cells were pretreated with YM3-PRF at concentrations ranging from of 0 to 20 μg/mL for 24 h. Then, the cells were exposed to LPS for 6 h and ATP for 30 min, respectively. The levels of IL-6 (**A**), IL-1β (**B**), and IL-18 (**C**) released into the culture supernatant were measured using ELISA, with the LPS + ATP-induced A549 cells serving as a reference set at 100%. The presented data represent the mean ± S.D. values obtained from three independent experiments. * *p* < 0.05, ** *p* < 0.01, and *** *p* < 0.001 indicate statistically significant differences compared with the LPS + ATP-induced control group.

To investigate the anti-inflammatory effects of YM3-PRF in LPS +ATP-induced A549 cells, the levels of proinflammatory cytokines (IL-6, IL-1β, and IL-18) released into the supernatant were measured using ELISA. We initially investigated the impact of YM3-PRF alone on cytokine release levels in A549 cells. The results revealed that the YM3-PRF-treated control group did not induce the IL-6, IL-1β, and IL-18 cytokine releases into the culture supernatant (Figure S1). The induction of LPS + ATP significantly increased the release of IL-6, IL-1β, and IL-18 when compared to the non-LPS + ATP group (*p* < 0.001). However, treatment with YM3-PRF resulted in a significant dose-dependent reduction in IL-6, IL-1β, and IL-18 releases from LPS + ATP-induced A549 cells (*p* < 0.001), as illustrated in Figure 2. These findings suggest that YM3-PRF exerts potent anti-inflammatory cytokine production in response to LPS + ATP stimulation.

### 3.5. Effect of YM3-PRF on Inhibition of IL-6, IL-1β, IL-18, and NLRP3 Gene Expressions in LPS + ATP-Exposed A549 Cells

**Figure 3:**
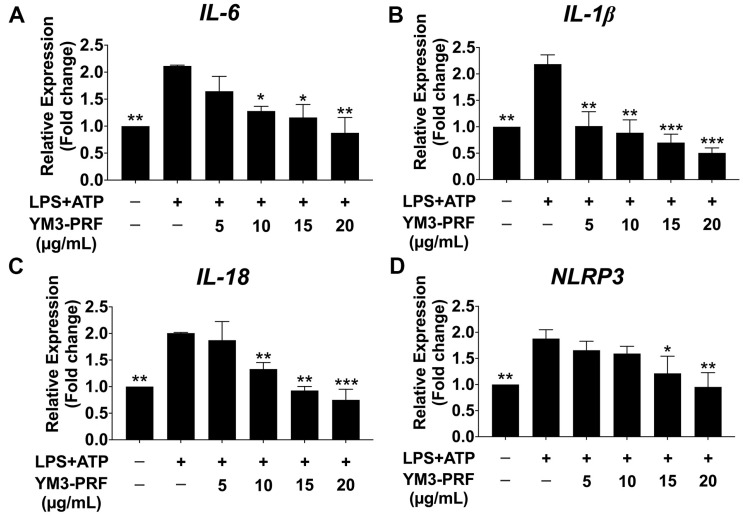
Inhibitory effects of YM3-PRF on NLRP3, IL-6, IL-1β, and IL-18 gene expression in LPS and ATP-induced A549 cells. A549 cells were pretreated with YM3-PRF at concentrations ranging from 0 to 20 μg/mL for 24 h. Then, the cells were exposed to LPS for 6 h and ATP for 30 min. The mRNA expressions of IL-6 (**A**), IL-1β (**B**), IL-18 (**C**), and NLRP3 (**D**) were assessed using RT-qPCR. The presented data represent the mean ± S.D. values obtained from three independent experiments; * *p* < 0.05, ** *p* < 0.01, and *** *p* < 0.001 indicate statistically significant differences compared to the LPS + ATP-induced A549 cells.

Previous studies have demonstrated that the NLRP3 inflammasome pathway, activated by LPS and ATP, contributes to the upregulation of proinflammatory cytokine gene expressions, including IL-6, IL-1β, IL-18, and NLRP3 [25]. In this study, we investigated the effects of YM3-PRF on the mRNA expressions of IL-6, IL-1β, IL-18, and NLRP3 in LPS + ATP-exposed A549 cells using RT-qPCR. Similar to the ELISA tests, the YM3-PRF-treated control group did not upregulate the IL-6, IL-1β, IL-18, and NLRP3 gene expressions (Figure S2). Figure 3 depicts that the mRNA levels of IL-6, IL-1β, IL-18, and NLRP3 were significantly increased in the LPS + ATP-induced A549 cells compared to the non-LPS + ATP group (*p* < 0.01). However, upon treatment with YM3-PRF, there was a significant dose-dependent reduction in IL-6, IL-1β, IL-18, and NLRP3 mRNA levels when compared to the LPS + ATP-induced A549 cells (*p* < 0.001). These results indicate that YM3-PRF exerts an anti-inflammatory effect by attenuating the expression of proinflammatory cytokine genes and NLRP3 in LPS + ATP-exposed A549 cells. The findings suggest that YM3-PRF may hold promise for further investigation as a potential inhibitor of the NLRP3 inflammasome pathway in the context of LPS + ATP-induced inflammation in A549 cells.

### 3.6. Inhibitory Effects of YM3-PRF on the NLRP3 Inflammasome Pathway in LPS + ATP-Induced A549 Cells

**Figure 4:**
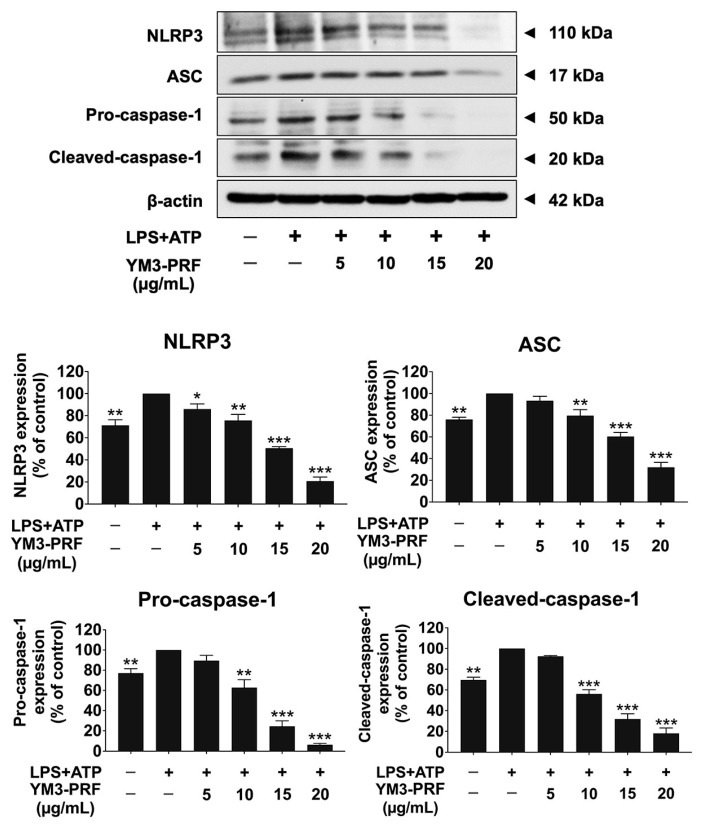
Inhibition of the NLRP3 inflammasome pathway in LPS + ATP-induced A549 lung cells by YM3-PRF. A549 lung cells were pretreated with YM3-PRF at concentrations ranging from 0 to 20 μg/mL for 24 h. Then, the cells were exposed to LPS for 6 h and ATP for 30 min. The data were visualized through Western blot analysis, and band density measurements were performed. The LPS + ATP-induced A549 cells are presented as 100% of the control. The presented data represent the mean ± S.D. value obtained from three independent experiments. * *p* < 0.05, ** *p* < 0.01, and *** *p* < 0.001 indicate statistically significant differences compared to the LPS + ATP-induced A549 cells.

The NLRP3 inflammasome pathway comprises NLRP3, ASC, pro-caspase-1, and cleaved-caspase-1, with its activation resulting in NLRP3 and ASC interaction. This interaction promotes the association of ASC with pro-caspase-1, leading to the activation of caspase-1 (cleaved caspase-1) and subsequent secretion of proinflammatory cytokines (IL-1β and IL-18) [15,21,26]. Therefore, inhibition of the NLRP3 inflammasome pathway holds promise as a potential target for combating LPS + ATP-induced inflammation. To investigate the effects of YM3-PRF on the NLRP3 inflammasome machinery, protein expressions were analyzed using Western blot in LPS + ATP-induced A549 cells. Figure 4 shows that the protein expressions of NLRP3, ASC, pro-caspase-1, and cleaved-caspase-1 were significantly elevated in LPS + ATP-induced A549 cells compared to the non-LPS + ATP group (*p* < 0.01). However, treatment with YM3-PRF resulted in a significant dose-dependent reduction in NLRP3, ASC, pro-caspase-1, and cleaved-caspase-1 protein expressions in LPS + ATP-induced A549 cells (*p* < 0.001). These findings suggest that YM3-PRF contributes to the anti-inflammatory properties in LPS + ATP-induced A549 cells by inhibiting the expressions of NLRP3 inflammasome machinery proteins. Consequently, this inhibition leads to a reduction in the secretion of proinflammatory cytokines (IL-1β and IL-18) at both gene and protein levels. Overall, YM3-PRF exhibits promising potential as an agent for mitigating inflammation by targeting the NLRP3 inflammasome pathway in A549 cells exposed to LPS + ATP.

### 3.7. Inhibitory Effects of YM3-PRF on NF-κB Nuclear Translocation in LPS + ATP-Induced A549 Cells

**Figure 5:**
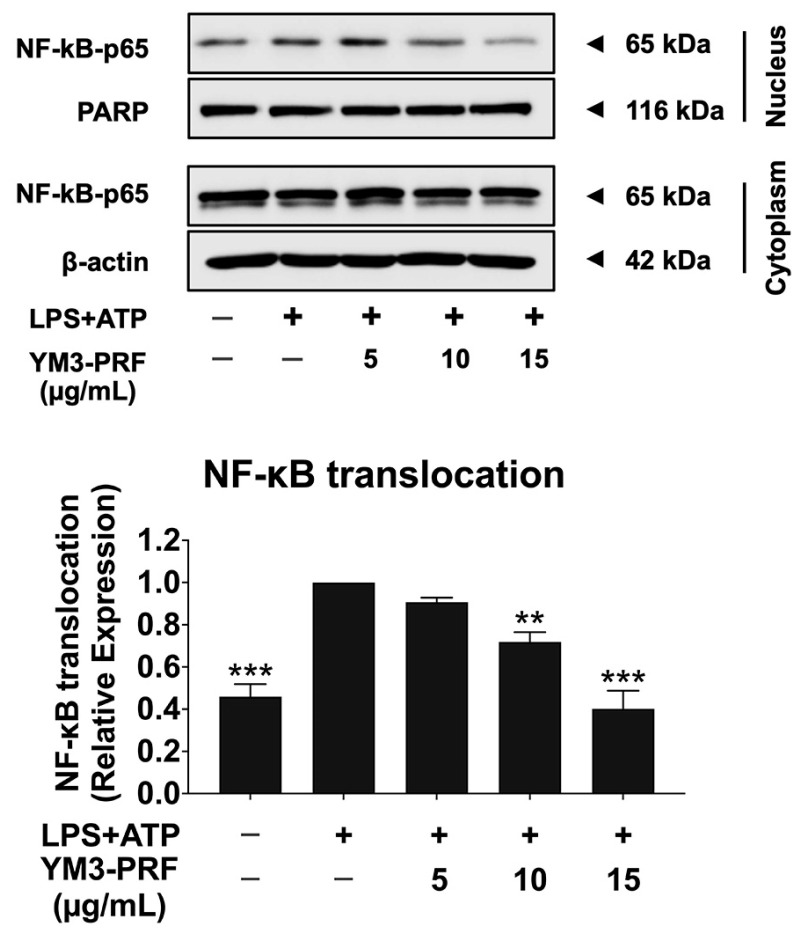
Inhibition of NF-κB nuclear translocation in LPS + ATP-induced A549 lung cells by YM3-PRF. A549 lung cells were pretreated with YM3-PRF at concentrations ranging from 0 to 15 μg/mL for 24 h. Then, the cells were exposed to LPS for 6 h and ATP for 30 min. The data were visualized through Western blot analysis, and band density measurements were performed. The LPS + ATP-induced A549 cells are presented as 100% of the control. The presented data represent the mean ± S.D. values obtained from three independent experiments. ** *p* < 0.01 and *** *p* < 0.001 indicate statistically significant differences compared to the LPS + ATP-induced A549 cells.

**Figure 6:**
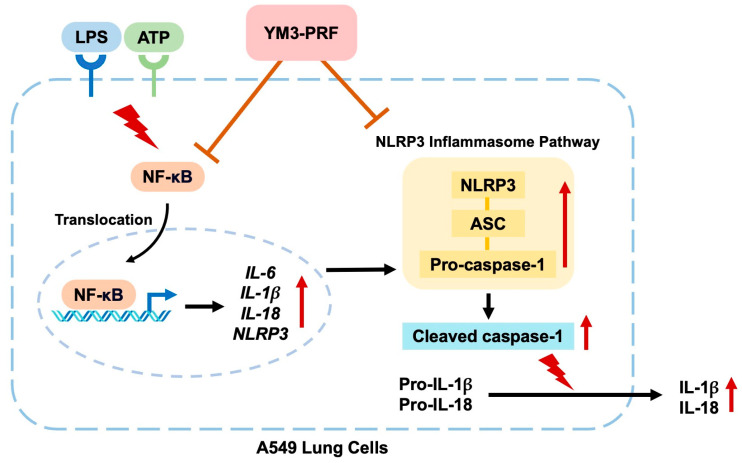
Schematic concluding mechanism of YM3-PRF attenuating LPS and ATP-induced NLRP3 inflammasome inflammation through inactivation of NF-κB nuclear translocation in A549 cells.

NF-κB is a transcription factor crucial for regulating genes involved in immune and inflammatory responses. Its activation and nuclear translocation lead to the induction of various proinflammatory mediators, including IL-6, IL-1β, and IL-18 [27]. In this study, we investigated the impact of YM3-PRF on NF-κB nuclear translocation in LPS + ATP-induced A549 cells using western blot analysis. Figure 5 illustrates that NF-κB-p65 protein expressions in the nucleus were significantly elevated in LPS + ATP-induced A549 cells compared to the non-LPS + ATP group (*p* < 0.01). However, treatment with YM3-PRF resulted in a significant dose-dependent inhibition of NF-κB-p65 protein expressions in the nucleus when compared to the LPS + ATP-induced group (*p* < 0.05). Conversely, there were no significant differences in NF-κB-p65 protein expressions in the cytoplasm. Overall, these findings indicate that YM3-PRF treatment effectively attenuates the LPS + ATP-induced NLRP3 inflammasome pathway by inhibiting NF-κB nuclear translocation. This inhibition subsequently leads to the suppression of inflammatory cytokine releases, including IL-6, IL-1β, and IL-18. Figure 6 illustrates the proposed mechanism by which YM3-PRF exerts its anti-inflammatory effects.

## 4. Discussion

Pigmented rice varieties display a diverse range of qualitative and quantitative traits, with their most distinctive feature being the presence of red, black, or purple pericarps. Many of these varieties are cultivated as traditional landraces in different regions worldwide. They have been discovered to possess higher nutritional qualities compared to conventional or commercial grain varieties. This is primarily attributed to their more efficient accumulation of nutraceuticals [28,29]. Among these pigmented rice varieties, Yamuechaebia 3 CMU stands out as a unique red jasmine rice strain derived through selective breeding of pure indigenous rice strains from high-altitude regions [29,30]. In this study, we utilized column chromatography with the Sephadex LH20 technique to isolate the proanthocyanidin-rich fraction (YM3-PRF) from the extract of red rice germ and bran. The extraction process followed a method previously described by us [31]. We evaluated the proanthocyanidins content in YM3-PRF using the vanillin assay. The total amount of proanthocyanidin in YM3-PRF (351.43 ± 1.18 mg/g extract) was found to be comparable to values reported in other relevant studies [14,31,32]. Phytochemicals, including proanthocyanidins, are naturally occurring compounds present in various plants and are well regarded for their environmentally friendly nature and safety profile. In the scope of our study, we assessed the cytotoxicity of the YM3-PRF extract on A549 cells. The results of the cytotoxicity analysis revealed that the YM3-PRF extract did not exhibit any cytotoxic effects on A549 cells. Furthermore, in terms of biocompatibility assessment, the YM3-PRF extract demonstrated noninductive properties towards hemolysis of red blood cells (RBCs) at the concentrations utilized in this study (Figure S3). As a consequence, the YM3-PRF extract emerged as a safe potential anti-inflammatory candidate for further investigation concerning its capacity to inhibit NLRP3 inflammasome pathways in A549 cells stimulated with LPS-ATP.

In this study, we investigated the anti-inflammatory properties of YM3-PRF using a monolayer A549 lung cell model induced by LPS + ATP. The A549 lung cells were chosen due to their frequent utilization in stimulating the inflammatory response triggered by the NLRP3 inflammasome activation [33,34]. Upon detecting a pathogen-associated molecular pattern (PAMP) or a danger-associated molecular pattern (DAMP), signaling cascades are initiated, leading to changes in gene expression, signal transduction, and cytokine production [35]. The NLRP3 inflammasome is implicated in various inflammatory conditions, including bacterial and viral lung infections, which can lead to excessive inflammation in alveolar macrophages and lung tissues. This uncontrolled inflammatory response is linked to a life-threatening condition called acute respiratory distress syndrome (ARDS), often resulting from a cytokine storm [36]. To establish the NLRP3 inflammasome inflammatory model in A549 lung cells, we employed a two-step priming process using LPS and ATP as the first and second signals, respectively. This classical NLRP3 inflammasome activation model has been widely employed in previous investigations [37,38].

The NLRP3 inflammasome pathway is recognized for triggering the production of specific cytokines, such as IL-1β and IL-18. These cytokines are released from lung cells to initiate the inflammatory response [34]. In our study, we observed that YM3-PRF effectively suppressed the inflammatory response in LPS + ATP-induced A549 cells by inhibiting the release of IL-1β and IL-18 cytokines. Red rice is distinguished by its rich content of proanthocyanidins, also known as oligomeric flavanols. These compounds make up over 60% of the total phytochemicals present in the rice seeds and their outer layer [39]. Proanthocyanidins are complex flavan-3-ol polymers primarily composed of catechin, epicatechin, gallocatechin, and epigallocatechin units. These compounds are also present in red rice germ and bran [13,28,40]. Proanthocyanidins belong to a class of flavonoids found in various plants, including grapes, berries, cocoa, and tea. They are renowned for their potent antioxidant properties and have been studied for potential health benefits, such as cardioprotection, anti-inflammatory activities, and anti-skin aging [41,42,43]. Previous research has indicated that medium polar fractions of red rice enriched with proanthocyanidins demonstrated robust anti-inflammatory activities by inhibiting the production of TNF-α, IL-6, and NO in LPS-activated macrophage. In contrast, the nonpolar fractions of red rice exhibited no anti-inflammatory properties [31]. Additionally, treatment with proanthocyanidin at concentrations of 1, 3, and 5 μg/mL significantly reduced the mRNA and protein levels of oxidative marker cyclooxygenase-2 (COX-2) and inducible nitric oxide synthase (iNOS), along with IL-6, IL-1β, and tumor necrosis factor-α (TNF-α) in LPS-induced bovine mammary epithelial cells [44]. Thus, it is plausible to attribute the inhibitory effects of YM3-PRF on LPS + ATP-induced A549 cells to the presence of proanthocyanidin in red rice germ and bran.

During infection, the lungs employ physical barriers and immune cells that release inflammatory cytokines like IL-6, IL-1β, and IL-18, initiating the inflammatory response. This process can be triggered through the NLRP3 inflammasome pathway, a cellular signaling mechanism [33,34]. The NLRP3 inflammasome pathway has been implicated in various respiratory diseases, including influenza, tuberculosis, *Streptococcus pneumoniae*, and *Staphylococcus aureus* infections [45,46,47,48]. At the molecular level, the presence of bacteria or cellular stress prompts cell damage, leading to the generation of PAMPs or DAMPs. These molecules stimulate inflammation by signaling through pattern-recognition receptors such as Toll-like receptors (TLRs) or nuclear oligomerization-domain-like receptors (NLRs). NLR molecules then form homo- or hetero-protein oligomers, activating the NF-κB transcription factor. This, in turn, initiates gene transcription, resulting in the production of mRNA expression for pro-IL-1β and pro-IL-18. The NLRP3 inflammasome complex, comprising effector subunit proteins NLRP3, ASC, and caspase-1, is assembled. This complex triggers the autocatalytic cleavage of pro-caspase-1, generating the active form of caspase-1 (cleaved-caspase-1). Caspase-1 further activates pro-IL-1β and pro-IL-18, converting them into IL-1β and IL-18, respectively. These proinflammatory cytokines are released from lung cells, triggering the inflammatory process. Subsequently, immune cells are attracted to the site of heightened inflammation, perpetuating the inflammatory response and potentially leading to unresolved or chronic inflammation [49,50]. In our study, we investigated the anti-inflammation mechanism of YM3-PRF using A549 lung cells. We found that YM3-PRF inhibited LPS + ATP-induced inflammation by impeding NF-κB nuclear translocation and downregulating the inflammasome-effector subunit proteins (NLRP3, ASC, and caspase-1), along with the active caspase-1 protein expression.

In summary, our research highlights the potential application of proanthocyanidin derived from red rice germ and bran as a viable therapeutic alternative for anti-inflammation purposes, particularly in the context of inflammatory lung diseases associated with NLRP3 inflammasome pathway activation. The data suggest that by inhibiting excessive inflammation through the suppression of the NLRP3 inflammasome pathway, which is responsible for the release of proinflammatory cytokines, it might be possible to mitigate lung tissue damage and hinder disease progression, including the development of conditions like lung fibrosis. However, it is crucial to acknowledge that while proanthocyanidin from red rice germ and bran has exhibited promising anti-inflammatory effects in this study, further investigations, encompassing in vivo experiments and clinical studies, are imperative to substantiate its potential as an effective anti-inflammatory agent.

## 5. Conclusions

This study demonstrates that the proanthocyanidin-rich fraction obtained from red rice germ and bran, specifically the YM3-PRF, exhibits promising anti-inflammatory properties against LPS and ATP-induced inflammation in A549 lung cells. The inhibition of inflammatory gene expressions and cytokine secretions suggested the potential of YM3-PRF as a functional food for preventing inflammatory conditions triggered by the activation of the NLRP3 inflammasome pathway. Importantly, YM3-PRF achieves its anti-inflammatory effects by inhibiting NF-κB nuclear translocation and downregulating NLRP3 inflammasome pathway machinery proteins. These results highlight the therapeutic potential of proanthocyanidin from red rice germ and bran as a prospective option for treating chronic inflammation diseases associated with NLRP3 inflammasome activation. Further studies are warranted to explore the efficacy of YM3-PRF in animal models and human trials, as well as to elucidate its underlying molecular mechanisms in greater detail.

## Figures and Tables

**Table 1:** The sequences of the primers used for qRT-PCR [22,23,24].

Gene Product	Primer Sequences
IL-6	Forward: 5′-ATG AAC TCC TTC ACA AGC-3′ Reverse: 5′-GTT TTC TGC CAG TGC CTC TTT G-3′
IL-1β	Forward, 5′-TGC TCA AGT GTC TGA AGC AG-3′ Reverse, 5′-TGG TGG TCG GAG ATT CGT AG-3′
IL-18	Forward, 5′-TCG GGA AGA GGA AAG GAA CC-3′ Reverse, 5′-TTC TAC TGG TTC AGC AGC CA-3′
NLRP3	Forward, 5′-AAC ATG CCC AAG GAG GAA GA-3′ Reverse, 5′-GGC TGT TCA CCA ATC CAT GA-3′
GAPDH	Forward, 5′-TCA ACA GCG ACA CCC AC-3′ Reverse, 5′-GGG TCT CTC TCT TCC TCT TGT G-3′

**Table 2:** Antioxidant activities of YM3-PRF.

	Antioxidant Capacity	
	IC_50_ ABTS Assay (μg/mL)	IC_50_ DPPH Assay (μg/mL)
YM3-PRF	1.75 ± 0.45 **	9.14 ± 1.01 **
Trolox	3.08 ± 0.22	
Vitamin E		33.92 ± 0.57

Data are presented as mean ± S.D. values of three independent experiments. ** *p* < 0.01 indicates statistically significant differences compared with the positive control (Trolox for ABTS assay and Vitamin E for DPPH assay).

## Data Availability

Not applicable.

## Supplementary Materials

The following supporting information can be downloaded at: https://www.mdpi.com/article/10.3390/nu15173793/s1. Figure S1. The effects of YM3-PRF on proinflammatory cytokine release in A549 cells. A549 cells were pretreated with YM3-PRF at concentrations ranging from of 0 to 20 μg/mL for 24 h. Then, the levels of IL-6, IL-1β, and IL-18 released into the culture supernatant were measured using ELISA. The presented data represent the mean ± S.D. values obtained from three independent experiments. Figure S2. The effects of YM3-PRF on NLRP3, IL-6, IL-1β, and IL-18 gene expression in A549 cells. A549 cells were pretreated with YM3-PRF at concentrations ranging from of 0 to 20 μg/mL for 24 h. Then, the mRNA expressions of NLRP3, IL-1β, and IL-18 were assessed using RT-qPCR. The presented data represent the mean ± S.D. values obtained from three independent experiments. Figure S3. The effect of YM3-PRF on normal red blood cells (RBCs) hemolysis. Red blood cells were treated with normal saline solution (NSS) (negative control), 1% TritonX-100 (positive control), 0–20 µg/mL of YM3-PRF for 4 h at 37 °C. Data are presented as mean ± S.D. values of three independent experiments. *** *p* < 0.001 compared to TritonX-100 (positive control).
