# Supplementary material for: Protective Effects of Proanthocyanidin-Rich Fraction from Red Rice Germ and Bran on Lung Cell Inflammation via Inhibition of NF-κB/NLRP3 Inflammasome Pathway
Source: Nutrients. 2023 Aug 30;15(17):3793. doi: 10.3390/nu15173793 (PMC10490275; doi:10.3390/nu15173793)
Supplement: Supplementary file 1 [file nutrients-15-03793-s001.zip › nutrients-2554199-supplementary.pdf]

Supplementary Materials

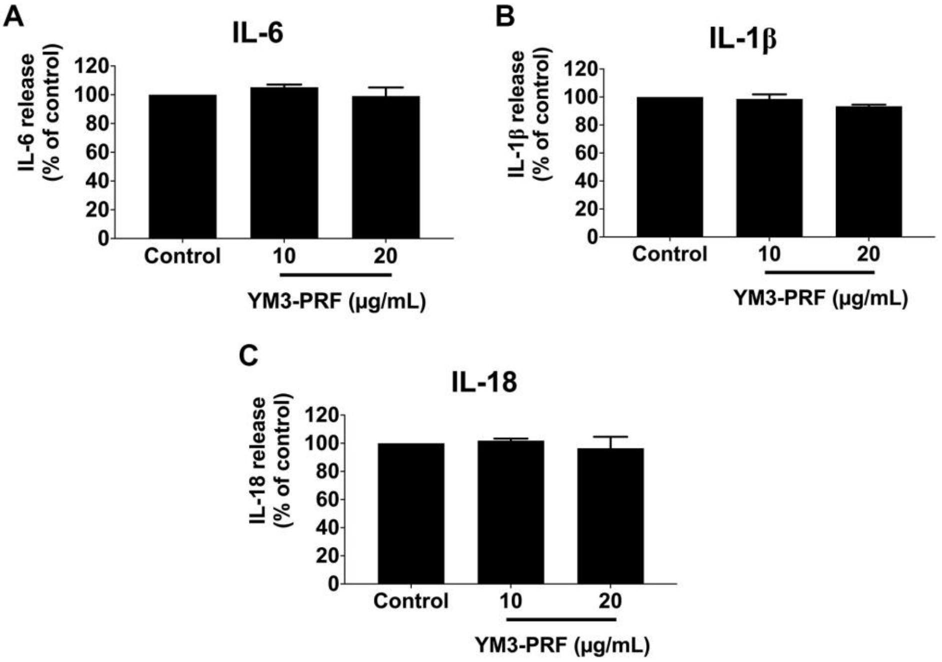

Figure S1. The effects of YM3-PRF on pro-inflammatory cytokine release in A549 cells. A549 cells were pre-treated with YM3-PRF at concentrations ranging from of 0 to 20 μg/mL for 24 h. Then, the levels of IL-6, IL-1β and IL-18 released into the culture supernatant were measured using ELISA. The presented data represent the mean ± S.D. values obtained from three independent experiments.

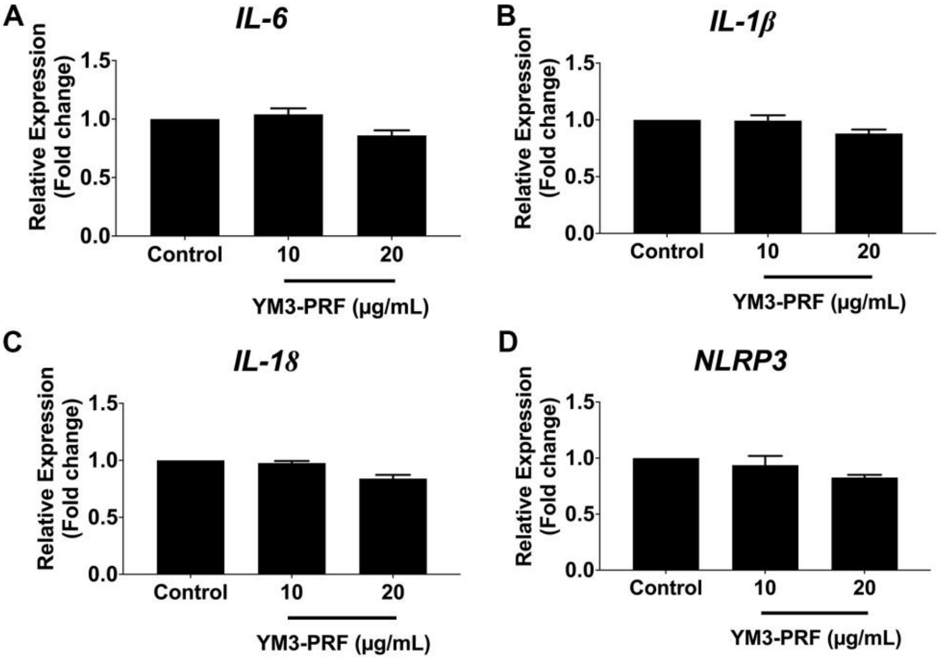

Figure S2. The effects of YM3-PRF on NLRP3, IL-6, IL-1β and IL-18 gene expression in A549 cells. A549 cells were pre-treated with YM3-PRF at concentrations ranging from of 0 to 20 μg/mL for 24 h. Then, the mRNA expressions of NLRP3, IL-1β and IL-18 were assessed using RT-qPCR. The presented data represent the mean ± S.D. values obtained from three independent experiments.

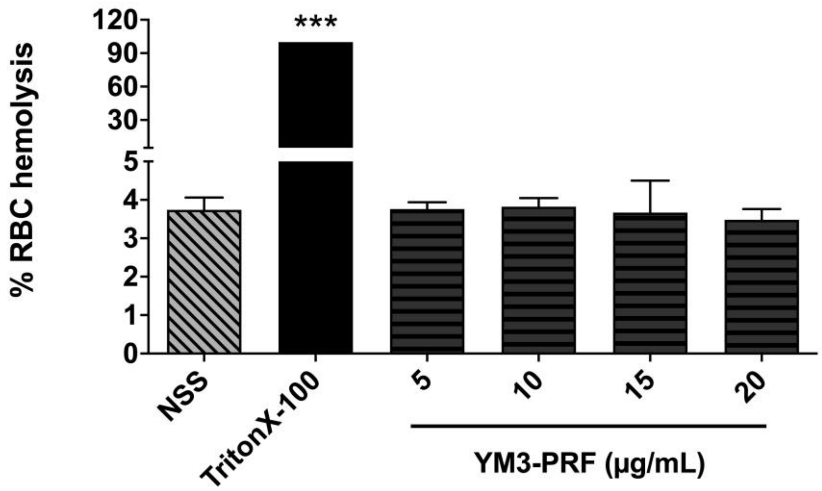

Figure S3. The effect of YM3-PRF on normal red blood cells (RBCs) hemolysis. Red blood cells were treated with normal saline solution (NSS) (negative control), 1% TritonX-100 (positive control), 0-20 µg/mL of YM3-PRF for 4 hours at 37°C. Data are presented as mean ± S.D. values of three independent experiments. *** $p < 0.001$ compared to TritonX-100 (positive control).
